# Azelaic Acid Versus Hydroquinone for Managing Patients With Melasma: Systematic Review and Meta-Analysis of Randomized Controlled Trials

**DOI:** 10.7759/cureus.41796

**Published:** 2023-07-12

**Authors:** Wardah Albzea, Rahf AlRashidi, Danah Alkandari, Moudhi Sadan, Abdulaziz Alkandari, Jaber J Alkanderi, Maisem T AlHajri, Saad N Almutairi, Athbi Alenzi, Shahad Alanazi, Safenaz Al-Qurashi, Raghad Alhajaji, Ahmad Al Shami

**Affiliations:** 1 Internal Medicine, Faculty of Medicine, Alexandria University, Alexandria, EGY; 2 Medicine and Surgery, Kuwait Institute for Medical Specializations, Kuwait City, KWT; 3 Emergency Department, Adan Hospital, Al-Ahmadi, KWT; 4 Pharmacology, College of Pharmacy, Alqassim University, Alqassim, SAU; 5 Family Medicine, Al-Awali Primary Health Care, Ministry of Health, Makkah, SAU; 6 Public Health Department, Makkah Health Affairs, Ministry of Health, Makkah, SAU; 7 Internal Medicine, New Jahra Hospital, Jahra, KWT

**Keywords:** systematic review and meta analysis, melasma area severity index, melasma, hydroquinone, azelaic acid

## Abstract

Melasma, a commonly acquired hyperpigmentation skin condition, is usually treated with topical agents as the first line of management. This systematic review and meta-analysis aimed to assess the efficacy and safety of azelaic acid versus hydroquinone in treating melasma patients. We conducted a comprehensive search across four online databases (PubMed, Scopus, Web of Science, and Cochrane Library) from the time of their creation until May 28, 2023. We considered randomized controlled studies comparing hydroquinone with azelaic acid for the treatment of melasma patients. We used the Cochrane Risk of Bias tool 2 to evaluate the risk of bias. The mean difference (MD) for continuous variables and the risk ratio (RR) for categorical variables, with a 95% confidence interval (CI) were pooled. Six studies were included, with a total of 673 patients with melasma. The azelaic acid had a lower mean change in melasma area severity index (MASI) than the hydroquinone group [MD= -1.23, 95% CI (-2.05, -0.40), P=0.004]. No difference was observed regarding the improvement via the objective response scale, the reduction in pigmentation, or the adverse events reported. However, despite not being statistically significantly different, there was a trend towards having more good responses in the azelaic acid group. Azelaic acid may be better than hydroquinone in reducing melasma severity (measured by MASI). However, larger studies with long-term follow-up are needed to validate these findings.

## Introduction and background

Melasma, a commonly acquired hyperpigmentation skin condition, is characterized by the appearance of brownish or greyish symmetrical patches on sun-exposed skin, most commonly on the face [1,2]. Women are more likely to be affected than males, and individuals with darker complexions are disproportionately affected [2,3]. Melasma, sometimes known as the "mask of pregnancy," is a common skin condition among pregnant women and those on hormonal birth control [3]. However, it is not limited to women who are pregnant or undergoing hormone replacement therapy as it can affect non-pregnant women, women who are not taking hormone replacement therapy, and also men [1-4]. Melasma is mostly a cosmetic problem that causes no physical pain or medical complications [5]. It may, however, have a substantial influence on a person's quality of life, causing emotional anguish, self-consciousness, and low self-esteem [5-7]. The visibility of pigmentation on the face may have an impact on social interactions and mental well-being [6].

The persistent and recurring nature of melasma presents substantial difficulties for patients and healthcare practitioners. Melasma is treated with a multimodal strategy that includes photoprotection, topical depigmenting treatments, chemical peels, laser therapy, and, in certain situations, oral drugs [8-11]. Sun protection is a critical component of melasma care [9]. In addition, a novel approach, platelet-rich plasma, which showed promising efficacy in certain conditions in the skin [12,13] and other systems [14,15] has been investigated in treating melasma and showed a promising effect [16].

Melasma is commonly treated with topical agents as the first line of management [9,17]. Hydroquinone, a depigmenting agent, has long been considered the gold standard due to its ability to inhibit melanin production [18-20]. Azelaic acid is a dicarboxylic acid that occurs naturally in grains such as wheat, rye, and barley. In treating melasma, it has multiple mechanisms of action [19,21]. Azelaic acid has been proven to suppress tyrosinase activity, diminish the formation of aberrant melanocytes, and have anti-inflammatory characteristics, making it an appealing alternative to melasma therapy [21-23].

Despite growing interest in azelaic acid and hydroquinone as melasma treatments, the comparative efficacy and safety of these medicines have not been comprehensively investigated. Some randomized controlled trials (RCTs) showed that azelaic acid is better than hydroquinone in treating melasma [24-26]; however, other studies showed the reverse [27,28]. Therefore, we carried out this systematic review and meta-analysis to assess the efficacy and safety of azelaic acid in comparison with hydroquinone in treating melasma patients.

## Review

Methods

We reported our systematic review and meta-analysis in accordance with the Preferred Reporting Items for Systematic Reviews and Meta-Analyses (PRISMA) declaration standards [29]. A thorough adherence to the Cochrane Handbook of Systematic Reviews and Meta-Analyses of Interventions was maintained throughout all processes [30]. Also, this study was registered in the International Prospective Register of Systematic Reviews (PROSPERO) database [ID: CRD42023433925].

Eligibility Criteria

Studies were considered for our assessment if they met the following requirements: (1) population - patients with melasma, (2) intervention - azelaic acid, (3) comparator: hydroquinone, (4) outcome - efficacy and safety outcomes. The efficacy outcomes included melasma area severity index (MASI), improvement via objective response scale, and reduction in pigmentation. The safety outcomes included local irritation, itching, and scaling.

Study Design

Controlled trials in which patients were randomly assigned to receive azelaic acid or hydroquinone. Both blind and open-label trials were taken into consideration. Studies with unreliable data for extraction and analysis, observational studies, those presented as theses or with abstracts only, studies for which complete full-texts were not accessible, case reports, case series, and review articles were all excluded.

Information Sources and Search Strategy

We performed a comprehensive search of four electronic databases (PubMed, Scopus, Web of Science, and Cochrane Central Register of Controlled Trials) from inception until May 28, 2023, using the following search strategy: ('melasma' or 'chloasma' or 'facial pigmentation' or 'melanoses') and ('azelaic acid' or 'Azelex' or 'Finacea' or 'skinoren' or 'monosodium azelate' or 'nonanedioic acid' or 'Dermaz 99' or 'Melazepam' or 'AzClear Action' or 'Azetec99' or 'Azepur99') and ('hydroquinone' or 'Quinol' or 'p-Dihydroxybenzenes' or 'para-Dihydroxybenzenes' or 'benzene-1,4-diol' or 'Melquin-3' or 'Solaquin' or 'Licoforte' or 'Fediquin' or 'Eldopaque' or 'Lustra'). Additionally, the listed papers' references were carefully checked for any other research that may have qualified.

Selection Process

The duplicates were eliminated, and the references were checked in two steps. In the first stage, the titles and abstracts of all recognized papers were checked independently by all authors to see if they were relevant to this meta-analysis. In the second phase, the full-text versions of the determined abstracts were checked to see if they were finally eligible for meta-analysis.

Data Collection Process, Data Items, and Risk of Bias

A standard data extraction sheet was used for data extraction. The data acquired included (1) characteristics of the included studies (study ID, country, design, total sample size, study arms, and dose regimen), (2) characteristics of the population of included studies [age (years), type of melasma, MASI, and previously treated patients], (3) risk of bias domains, and (4) outcome measures (efficacy outcome: MASI, improvement via objective response scale, and reduction in pigmentation; safety outcomes: local irritation, itching, and scaling). We used the Cochrane assessment tool 2 (ROB2) for randomized controlled trials [31]. The risk of bias assessment included the following domains: bias arising from the randomization process, bias due to deviations from intended interventions, bias due to missing outcome data, bias in the measurement of the outcome, bias in the selection of the reported result, and other biases. The authors' judgments are categorized as 'low risk', 'high risk', or 'some concerns' of bias.

Effect Measures

In the present meta-analysis, we considered the following outcome measures:

1. MASI is used to assess the severity of melasma and the changes during the therapy: The MASI scoring system takes into account four main parameters: the darkness (pigmentation intensity) of the melasma patches, the homogeneity (uniformity) of the pigmentation, the extent (area) of the affected skin, and the location of the patches on the face. Each parameter is assigned a score based on its severity, and the scores are then summed to obtain a total MASI score for an individual. Higher MASI scores indicate more severe melasma, with a maximum score of 48. MASI is presented as mean and standard deviation, so the effect measure in this study is presented as the mean difference (MD) between the two groups.

2. Improvement via objective response scale: This scale divides the patients according to the response rate into four classes (excellent, good, fair, and poor). The number of patients in each category at the end of the study was compared between the two groups (by event and total in each group).

3. Reduction in pigmentation: The pigmentary intensity of melasma was compared with the non-affected parts of the skin of the face; then, the pigmentation of the melasma was rated on a five-point scale (1 = no difference, 5 = intensely more pigmented). The reduction of the pigmentation was evaluated according to the level of reduction (reduction by one level, reduction by two levels, and reduction by three levels or more). The number of patients in each category at the end of the study was compared between the two groups (by event and total in each group).

4. Adverse events (local irritation, itching, and scaling): The adverse events incidence was compared between the two groups (by event and total in each group).

Synthesis Methods

The dichotomous data were reported as risk ratios (RR), while the continuous data were presented as mean differences (MD) for the azelaic acid and hydroquinone groups. Review Manager 5 [(RevMan 5) (Computer program) Version 5.4. Copenhagen: The Cochrane Collaboration, 2020] was used to conduct a DerSimonian Liard meta-analysis [32] of the RR or MD and their associated 95% confidence intervals. P-values lower than 0.05 were regarded as statistically significant. We calculated the pooled effect size for each outcome using the DerSimonian Liard meta-analysis approach [32]. This random effect model, which assumes that the included studies represent a random sample from the population, provides considerably greater weight to small studies compared to the expenditures of larger research. This model was chosen because, in opposition to the fixed-effects model, it permits a bigger standard error in the pooled estimate, making it suitable in the case of contradictory or disputed estimates. As a consequence, the impacts that our meta-analysis identified are conservative estimates that take any inconsistencies into consideration. The Chi-square test (Cochrane Q test) evaluated statistical heterogeneity among studies. Then, the chi-square statistic, Cochrane Q, was used to calculate the I-squared according to the equation: I2= ( Q−df Q ) x100%. A chi-square P value less than 0.1 was considered significant heterogeneity. I-square values ≥50% were indicative of high heterogeneity. Since Egger and colleagues [33,34] claim that publishing bias evaluation is inaccurate for less than 10 pooled studies, we were unable to determine the presence of publication bias in the current research using Egger's test for funnel plot asymmetry.

Results

Literature Search Results

**Figure 1:**
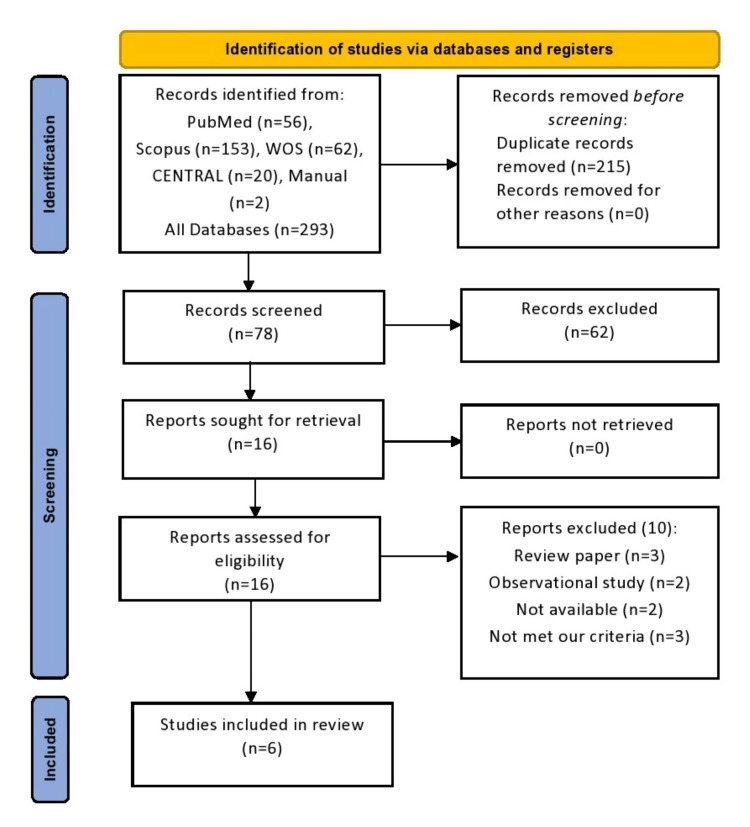
PRISMA flow chart for the selection process. [24-28, 35]

Our literature search strategy turned up a total of 293 documents after eliminating the duplicates. The screening of the titles and abstracts of the papers resulted in the identification of sixteen articles that might proceed to the full-text screening. Six of them have been included in the meta-analysis. No further papers were included after manually searching the references of the listed studies. The PRISMA flow diagram of the study selection process is shown in Figure 1.

Characteristics of the Included Studies

**Figure 2:**
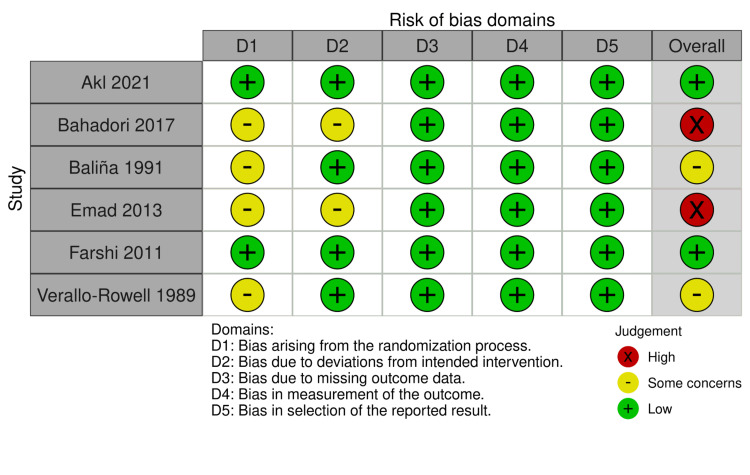
Risk of bias graph of the included studies. [24-28, 35]

Thirteen studies were included in the meta-analysis [24-28,35], with a total of 673 patients with melasma. In all studies, patients were assigned to receive either azelaic acid or hydroquinone. The majority of patients were middle-aged females, and epidermal melasma was the most frequent type. Most of the included studies used the drug once at night in addition to sunscreen. A summary of the characteristics of the included studies is provided in Table 1, and the baseline characteristics of the population of the included studies are presented in Table 2. Overall, the risk of bias was low in two studies, with some concerns in two studies, and high in two studies, according to the Cochrane Risk of Bias tool 2, Figure 2. Four RCTs [25-28] were evaluated as having some concerns in the randomization process domain because they did not provide any information regarding the randomization and allocation concealment process. However, two RCTs [26,28] were evaluated as having some concerns in deviation from the intended treatment domain because there is no information on whether deviation from usual practice will affect the outcomes. 

**Table 1:** Summary of the included studies. [24-28, 35]

Study ID	Country	Design	Total sample size	Study arms		Dose regimen
				Intervention	Control	
Alk [24]	Egypt	RCT	n=50	Azelaic acid 20%	Hydroquinone 4%	For 15 min daily before bedtime for three months, plus oral tranexamic acid.
Bahadori et al. [26]	Iran	RCT	n=44	Azelaic acid 20%	Hydroquinone 4%	Once nightly, and throughout the day + a non-greasy sunscreen with SPF 28 for 4 months.
Baliña et al. [25]	Multi-central	RCT	n=329	Azelaic acid 20%	Hydroquinone 4%	Twice daily + broad-spectrum sunscreen for 24 weeks.
Emad et al. [28]	Iran	RCT	n=66	Azelaic acid 20%	Hydroquinone 4%	Every night, azelaic acid to the left side and hydroquinone to the right side + sunscreen with SPF 30 every 3 hours for 20 weeks.
Farshi [35]	Iran	RCT	n=29	Azelaic acid 20%	Hydroquinone 4%	Twice daily + broad-spectrum sunscreen every 3 h for 8 weeks
Verallo-Rowell et al. [27]	Philippines	RCT	n=155	Azelaic acid 20%	Hydroquinone 2%	Twice daily for 24 weeks + broad-spectrum sunscreen

**Table 2:** Baseline characteristics of the included studies. [24-28, 35]

Study ID	Group	Sample size	Previously treated	Age (years)	Type of melasma			Melasma Area Severity Index (MASI)
					Epidermal	Dermal	Mixed	
Alk [24]	Azelaic acid 20%	n=25	NR	36.56 (5.42)	9 (36%)	6 (24%)	10 (40%)	17.06 (1.51)
	Hydroquinone 4%	n=25	NR	35.2 (4.51)	10 (40%)	7 (28%)	8 (32%)	17.77 (1.45)
Bahadori et al. [26]	Azelaic acid 20%	n=23	NR	NR	NR	NR	NR	8.92 (1.02)
	Hydroquinone 4%	n=21	NR	NR	NR	NR	NR	8.64 (0.83)
Baliña et al. [25]	Azelaic acid 20%	n=164	71 (43.3%)	35 (18-57)	122 (74.7%)	NR	41 (25.3%)	NR
	Hydroquinone 4%	n=165	78 (47.3%)	34(21-41)	117 (71.2%)	NR	47 (28.2%)	NR
Emad et al. [28]	Azelaic acid 20%	n=33	NR	32.7 (6.4)	NR	NR	NR	7.88 (3.27)
	Hydroquinone 4%	n=33	NR	32.7 (6.4)	NR	NR	NR	7.8 (3.36)
Farshi [35]	Azelaic acid 20%	n=14	NR	34.6 (6.6)	14 (100%)	0 (0%)	0 (0%)	7.6 3.5)
	Hydroquinone 4%	n=15	NR	34.6 (6.6)	15 (100%)	0 (0%)	0 (0%)	7.2 (3.2)
Verallo-Rowell et al. [27]	Azelaic acid 20%	n=77	37 (48.1%)	NR	43 (55.8%)	0 (0%)	34 (44.2%)	NR
	Hydroquinone 2%	n=78	41 (52.6%)	NR	38 (48.7%)	0 (0%)	39 (50%)	NR

MASI

**Figure 3:**
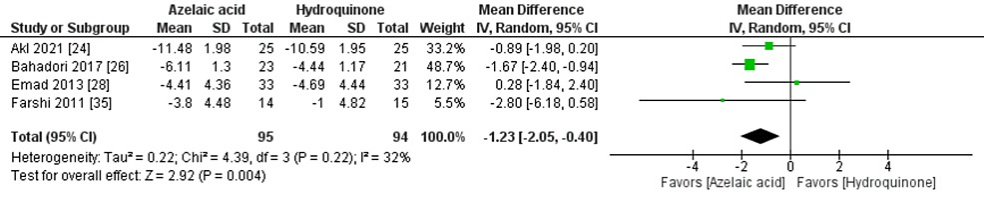
Meta-analysis of the mean change of Melasma Area Severity Index (MASI).

The overall MD of MASI favored the azelaic acid group over the hydroquinone group as the improvement was statistically significant and more obvious in the azelaic acid group [MD= -1.23, 95% CI (-2.05, -0.400, P=0.004], Figure 3. The pooled studies were homogenous (P=0.22; I2=32%).

Improvement via Objective Response Scale

**Figure 4:**
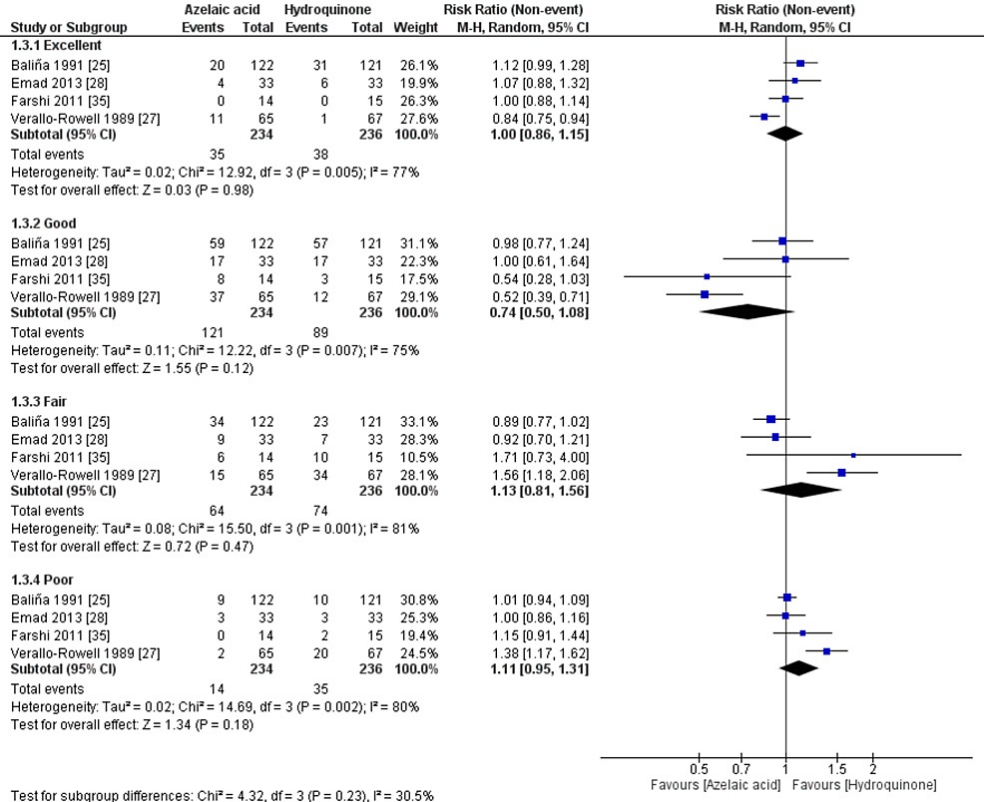
Meta-analysis of the improvement via objective response scale.

The overall RR for Improvement via the objective response scale did not favor either of the two groups. For excellent objective response, [RR= 1.00, 95% CI (0.86 to 1.15), P=0.98]; for a good objective response, [RR= 0.74, 95% CI (0.50 to 1.08), P=0.12]; for a fair objective response, [RR= 1.13, 95% CI (0.81 to 1.56), P=0.47]; and for poor objective response, [RR= 1.11, 95% CI (0.95 to 1.31), P=0.18], Figure 4. However, there was a trend towards having more good responses in the azelaic acid group and more poor responses in the hydroquinone group, despite being non-statistically significant. The pooled studies were not homogenous for all response subgroups, with significantly high heterogeneity (P<0.1; I2>50%).

Reduction in Pigmentation

**Figure 5:**
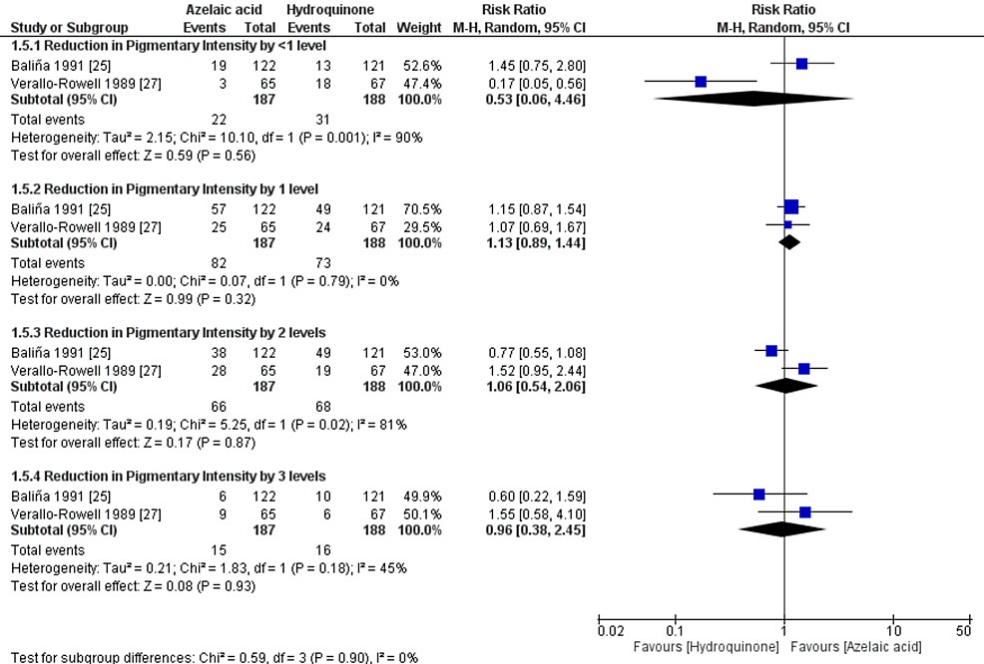
Meta-analysis of reduction in pigmentation.

The overall RR for the reduction in pigmentation did not favor either of the two groups for all subgroups. For reduction in pigmentation by < one level, [RR= 0.53, 95% CI (0.06 to 4.46), P=0.56]; for the reduction in pigmentation by one level, [RR= 1.13, 95% CI (0.89 to 1.44), P=0. 32]; for the reduction in pigmentation by two levels, [RR= 1.06, 95% CI (0.54 to 2.06), P=0.87]; and for the reduction in pigmentation by three levels, [RR= 0.96, 95% CI (0.38 to 2.45), P=0.93], Figure 5. The pooled studies were not homogenous for all subgroups, with significant high heterogeneity (P<0.1; I2>50%), except for the reduction in pigmentation by one level subgroup, which was homogenous (P=0.79; I2=0%).

Adverse Events

**Figure 6:**
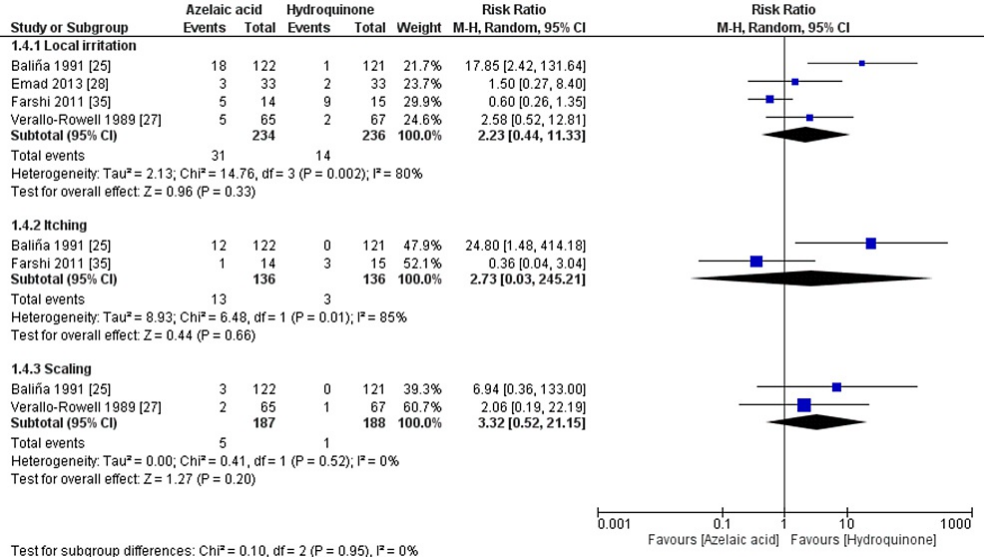
Meta-analysis of adverse events after treatment.

The pooled RR for the adverse events did not favor either of the two groups, Figure 6. For local irritation, [RR= 2.23, 95% CI (0.44 to 11.33), P=0.33]; for itching, [RR= 2.73, 95% CI (0.03 to 245.21), P=0.66]; and for scaling, [RR= 3.32, 95% CI (0.52 to 21.15), P=0.20}, Figure 6. The pooled studies were not homogenous for all, with significant high heterogeneity (P<0.1; I2>50%), except for the scaling subgroup, which was homogenous (P=0.52; I2=0%).

Discussion

Significance of the Study and Summary of Findings

To the best of our knowledge, this is the first systematic review and meta-analysis that compares azelaic acid and hydroquinone in treating patients with melasma. The superiority of azelaic acid or hydroquinone in treating melasma has been the subject of conflicting research in the past. Therefore, performing a thorough meta-analysis can enhance understanding of the information at hand and guide clinical judgment. Our analysis of the six included studies revealed that azelaic acid outperformed hydroquinone in terms of improving MASI scores. However, there was no discernible difference between the two treatment groups when we looked at the objective response scale and pigmentation decrease, despite the existence of a trend towards having more good responses in the azelaic acid group and more poor responses in the hydroquinone group. In addition, the two interventions were comparable regarding the adverse events reported.

Explanation of the Finding

The recent evidence suggests that networks of cellular interactions between keratinocytes, fibroblasts, and mast cells play a key role in the melasma [36] and that cumulative sun exposure leads to a change in pigmentation in a microenvironment of cutaneous photoaging in which inflammatory cells, particularly mast cells, infiltrate the dermis [36-38]. Therefore, azelaic acid, which was used as an adjuvant agent in treating melanoma because of its cytotoxic and anti-proliferative properties on the abnormal melanocytes [39,40], is also considered a depigmenting drug in melasma [41,42]. By inhibiting the tyrosinase activity, azelaic acid diminishes the formation of aberrant melanocytes, and it also has anti-inflammatory characteristics, making it an appealing alternative for melasma therapy [21-23] because the inflammatory cells and inflammation play a crucial role in the pathogenesis of melasma [37,38].

On the other hand, since 1950, hydroquinone has been a component of over-the-counter skin-lightening cosmetics, and since 1960, it has also been a component of medicinal products [43,44]. Because of side effects such as leukoderma-en-confetti, occupational vitiligo, and exogenous ochronosis, hydroquinone has been prohibited from use in cosmetic skin-lightening formulations in countries of the European Union since 2001 [45,46]. However, recent evidence indicates that additional potential long-term impacts, like carcinogenesis, may also be anticipated [47]. The majority of carcinogenesis is caused by hydroquinone metabolites that are produced in the liver [47]. Although no research has yet shown that its application to the skin causes carcinogenesis, we should be aware of this possible risk [45-47].

Although hydroquinone primarily acts by suppressing tyrosinase activity, it has little effect on inflammation [48]. Therefore, azelaic acid's dual mode of action may give a more comprehensive treatment approach for melasma, with better results than hydroquinone alone, as observed in this study.

Implications of These Findings in Practice

The clinical ramifications of our findings are significant. To begin, azelaic acid's higher efficacy in improving MASI scores implies that it may be an effective treatment option for people with melasma. Clinicians may add azelaic acid into their treatment protocols as a monotherapy or in conjunction with other medications, such as oral tranexamic acid [24], depending on the needs and features of the individual patient. Second, the finding that azelaic acid has a better safety profile than hydroquinone suggests that it may be especially suitable for individuals susceptible to skin irritation or concerned about hydroquinone's potential adverse effects.

Strength Points and Limitations

This research has various strengths, including the fact that it is the first and most comprehensive meta-analysis comparing the safety and effectiveness of azelaic acid to hydroquinone. In order to verify that the articles selected for our analysis were of high quality and comparable, we also used stringent inclusion and exclusion criteria. Since we included studies that were published in all languages and translated them into English before collecting the data, we did not restrict the language of the published publications either. This meta-analysis does have some limitations, most notably the very small sample sizes of the included publications (only six), which may restrict the generalizability of our results. Additionally, not all trials reported every relevant outcome.

Recommendations for Future Research

First, conducting well-designed RCTs with bigger sample sizes will give more robust evidence of the efficacy and safety of azelaic acid over hydroquinone in treating melasma. These studies should also attempt to include a different patient population to improve the generalizability of the results. Second, long-term follow-up studies are required to assess the durability of therapy effects as well as the possibility of relapse. Furthermore, more studies should be conducted to determine the best treatment duration and maintenance measures for sustaining the effects of azelaic acid therapy. Finally, combining azelaic acid with additional drugs or techniques or comparing it with other therapies of melasma, not only hydroquinone, may provide valuable insights into potential synergistic effects and improve treatment outcomes in melasma.

## Conclusions

We conclude that azelaic acid may be better than hydroquinone in reducing melasma severity as measured by the MASI. However, no significant difference between both treatments regarding the side effects. To validate these results and optimize treatment regimens for melasma, however, further study is necessary with bigger sample sizes and long-term follow-up. When choosing therapy for melasma, clinicians should consider the patient's traits and preferences in addition to the treatment's effectiveness, safety, and possible long-term results.
